# A review of the effects of *Urtica dioica *(nettle) in metabolic syndrome

**DOI:** 10.22038/IJBMS.2022.58892.13079

**Published:** 2022-05

**Authors:** Bahareh Samakar, Soghra Mehri, Hossein Hosseinzadeh

**Affiliations:** 1 Department of Pharmacodynamics and Toxicology, School of Pharmacy, Mashhad University of Medical Sciences, Mashhad, Iran; 2 Pharmaceutical Research Center, Pharmaceutical Technology Institute, Mashhad University of Medical Sciences, Mashhad, Iran

**Keywords:** Diabetes, Hyperlipidemia, Hypertension, Metabolic syndrome, Nettle, Urtica dioica

## Abstract

Metabolic syndrome is a serious health condition, yet a common worldwide disorder. It includes several risk factors such as hypertension, dyslipidemia, and high glucose levels which lead the patients to higher risks of cardiovascular diseases, diabetes, and stroke. Phytotherapy plays an important role in treating components of metabolic syndrome. Nettle (*Urtica dioica*) is considered a valuable plant due to bioactive compounds such as formic acid and rich sources of flavonoids. To acknowledge the role of nettle in metabolic syndrome, several mechanisms have been suggested such as alterations in potassium and calcium channels which improve hypertension. Antihyperlipidemic properties of nettle are mediated by inhibition of HMGCoA reductase and amelioration of lipid peroxidation via antioxidant effects. Also, one of the flavonoids in nettle, quercetin, is responsible for decreasing total cholesterol. Moreover, nettle is responsible for anti-diabetic effects through processes such as increasing insulin secretion and proliferation of pancreatic β-cells. This review aims to gather different studies to confirm the potential efficacy of nettle in metabolic syndrome.

## Introduction

There are various definitions of metabolic syndrome by different healthcare organizations. According to WHO, metabolic syndrome is a pathologic condition characterized by hypertension, hyperlipidemia, insulin resistance, and abdominal obesity. This syndrome could lead to diseases like type 2 diabetes, stroke, coronary diseases, and other disabilities (1). A wide variety of herbal extracts and their active components could be used for controlling metabolic syndrome (2). Some of these plants and their active constituents include *Allium sativum *(garlic) (3), *Nigella sativa *(black seed) and its active constituent, thymoquinone (4), *Vitis vinifera* (grapes) (5), *Crocus sativus* (saffron) (6), *Cinnamomum verum* (cinnamon) (7), *Capsicum annuum *and its constituent, capsaicin (8), *Berberis vulgaris* (barberry) (9), *Persea americana* (avocado) (10), *Silybum marianum* (milk thistle) (11), *Abelmoschus esculentus *(okra) (12), *Crataegus pinnatifida *(Chinese hawthorn) (13), and *Rosmarinus officinalis* (rosemary) (14) which exhibit significant effects in the management of the metabolic syndrome.

Stinging nettle (*Urtica dioica *L.) from the *Urticaceae* family, grows in the wild form in North Africa, North America, Europe, and Asia (15). Its derivatives including crude dried powder, infusion (herbal tea), dry extract, decoction, or fresh juice are considered unquestionably important in phytotherapy (16). Nettle contains various biochemicals such as formic acid, histamine, and acetylcholine (17) and valuable compounds like flavonoids, tannins, phytosterols, saponins, proteins, and amino acids (18). Flavonoids present in nettle include flavonols, flavanones, and flavonoid glycosides. The water and alcoholic extracts of aerial parts of nettle contain vitamins such as thiamine, riboflavin, pyridoxine, folic acid, nicotinic acid, and ascorbic acid (19). Studies on the whole nettle herb have resulted in the isolation of several compounds: quercetin, trans-ferulic acid, beta-sitosterol, erucic acid, dotriacotane, ursolic acid, scopoletin, rutin, and p-hydroxylbenzalcohol (20). GC–MS analysis of nettle’s  essential oil identified 43 compounds and revealed that the main components are carvacrol (38.2%), carvone (9.0%), naphthalene (8.9%), (E)-anethol (4.7%), hexahydrofarnesyl acetone (3.0%), (E)-geranyl acetone (2.9%), (E)-β-ionone (2.8%), and phytol (2.7%) (21).

Nettle has been used since antiquity, as it is one of the most appreciated domestic plants of Dioscorides, “the father of pharmacognosy” (22). According to various studies, *U. dioica *is a plant with so many therapeutic potential effects in different disorders such as prostatic hyperplasia (23), arthritis rheumatoid (24), allergies (25), anemia (26), internal bleeding (27), kidney stones (28), and burns (29). Furthermore, it has anti-proliferative (30) and antimicrobial activity and has been proven to cure infectious diseases (31).

Diabetes is one of the risk factors leading to metabolic syndrome. Nearly 5% of the world’s population is suffering from this chronic disorder (32). Nettle’s consumption could have an effective role in type 2 diabetes by several mechanisms such as increasing glucose uptake by skeletal muscles and adipose tissues (33) and its anti-inflammatory activities (34). Moreover, the use of *U. dioica *as an anti-oxidant could be an effective approach to control diabetes and reduce associated complications (35). 

The second risk factor for metabolic syndrome is increasing lipid profile levels. Cardiac morbidity and mortality are directly related to hyperlipidemia and hypercholesterolemia which are important coronary risk factors. It has been shown that *U. dioica *can be useful in this matter by decreasing lipid peroxidation and liver enzyme activity (36). The last but not less important risk factor for metabolic syndrome is hypertension which affects one billion people across the world and takes nine million lives every year. Medicinal plants such as *U. dioica *have been proven beneficial to the cardiovascular system and could be used in hypertension therapy (37, 38). Thus evidently, nettle has a potential role in decreasing the three major risk factors for metabolic syndrome which are hypertension, hyperlipidemia, and hyperglycemia. Industrial lifestyle and lack of exercise needed in a daily routine, have increased the number of people suffering from cardiovascular problems. While current chemical treatments have not been able to cease this ever-growing problem, natural products are considered vastly important in the treatment of the metabolic syndrome. Several investigations have studied the effect of *U. dioica *in the treatment of different aspects of metabolic syndrome separately but there are no comprehensive studies to collect this information. Therefore, in this review, the most relevant studies to evaluate the role of *U. dioica *in metabolic syndrome with a special focus on underlying mechanisms were described.

## Methods

In this review article, different electronic databases or search engines such as Pubmed, Scopus, and Google Scholar have been used to search with the following keywords: *U. dioica*, nettle, metabolic syndrome, hypertension, blood pressure, dyslipidemia, hypercholesterolemia, hyperlipidemia, hyperglycemia, and diabetes. We collected all published *in vitro*, *in vivo*, and clinical studies investigating the effects of *U. dioica *on metabolic syndrome. The most relevant articles were included without publication time limitation.

**Anti-hypertensive activity**

Hypertension is a risk factor for premature cardiovascular diseases. Therefore it could lead to excessive morbidity and mortality (39). Estimates suggest that the prevalence of hypertension has increased drastically in the past few years especially in middle and low-income countries. However, the levels of awareness and controlling this ever-growing disease are low. Therefore, hypertension plays an important role in the pathogenesis of cardiovascular diseases (40). Herbs contain many phytochemicals that prove to be effective in inducing moderate reductions in blood pressure either alone or in combination with current antihypertensive drugs. Therefore their use continues to increase in popularity in both developing and developed countries (41). *U. dioica *has been used as an anti-hypertensive remedy for many years. Nettle extract could improve cardiac performances by decreasing both systolic and diastolic blood pressure (37). To observe the effect of extracts and fractions, several experiments have been performed.

**Animal studies**

Various studies have attempted to explain the positive effect of nettle on hypertension through animal featuring experiments. One is an experiment in which model rats received continuous intravenous perfusion of aqueous extract of nettle for 1.25 hr at a low dose (4 mg/kg/hr), a high dose (24 mg/kg/hr), and control diuretic (2 mg/kg/hr furosemide) respectively. As a result, there was a reduction in arterial blood pressure proportionally to the dosage of plant extract. Also, an increase in diuresis and natriuresis was observed (38). In a 4-week experiment, Vajic *et al*. supplemented hypertensive rats with 10, 50, and 200 mg/kg/day of nettle extract and 10 mg/kg/day of losartan. At the end of the trial, all three test groups showed a reduction in cardiac index, systolic, and diastolic blood pressure. Furthermore, the plasma anti-oxidant capacity was improved and the systematic oxidative stress was reduced (37). In a survey conducted by Qayyum *et al*., the crude methanolic extract of nettle and its fractions were tested on model rats (normal or hypertensive) under anesthesia to examine their blood pressure-lowering effect. The subjects were injected intravenously with 0.1 ml saline or with the same volume of test substances. As a result, both normotensive and hypertensive rats indicated a dose-dependent fall in their mean arterial pressure with a more significant response in the hypertensive ones. Among the different fractions of extract tested, the aqueous fraction was the least potent whereas the ethyl acetate fraction was the most potent. The thoracic aorta from normotensive and high salt-induced hypertensive rats was cut into rings 2–3 mm wide. The extract of nettle and its fractions were cumulatively added into the organ bath. Force and pressure transducers were connected to the PowerLab Data Acquisition System to identify the mechanisms of vasorelaxation. As the final result, the extract of nettle showed antihypertensive effects through calcium channel blocking effects and nitric oxide (NO)-mediated vasorelaxation (42). The aqueous extract of nettle (1 and 2 g/l) could cause dose-dependent bradycardia in a rat’s isolated heart which leads to a hypotension effect. Surprisingly nettle extract induces vasoconstriction of rat’s isolated aorta through activation of α_1_-adrenergic receptors causing elevated blood pressure. However, this effect may differ in small vessels. Vasoconstriction-induced hypertension may be masked *in vivo* by the bradycardia, therefore only the hypotensive effect of nettle extract is observed (43).

**Human studies**

Many studies worldwide have examined phytotherapeutic medicines among hypertensive patients, such as the study done by Ziyyat *et al*. This examination shows 67.5% of patients use medicinal plants regularly. For treating hypertension, 18 plants were cited, of which the most used were *U. dioica *L. (Urticaceae), *A. sativum* L. (Liliaceae), *Arbutus unedo* L. (Ericaceae), *Olea europaea *L. (Oleaceae), and *Petroselinum crispum* A.W. Hill (Apiaceae) (44). In a placebo-controlled, randomized study with hypertensive subjects, the effect of three different herbal extracts (*U. dioica, Mentha longifolia*, and *Viola odorata*) was investigated. Systolic and diastolic blood pressures were monitored during the treatment with 300 ml/day of plant extracts for 16 weeks. Results revealed the dose- and duration-dependent significant reductions in blood pressures of subjects treated with either of three extracts (45). Via a randomized, single-blind, clinical trial which was performed on type 2 diabetic patients, the intervention group was treated daily with hydroalcoholic extract of nettle (100 mg/kg/day). As a result, it was observed that after 8 weeks, the systolic blood pressure was significantly reduced. Therefore, Tarighat *et al*. has concluded that the hydroalcoholic extract of nettle could be a suitable auxiliary therapy for type 2 diabetic patients due to its positive effects on the blood pressure status (46). A study with 40 diabetic men indicated that aerobic exercise and nettle supplement (10 g/day) were both effective on diastolic blood pressure after 8 weeks of trial. Also, there was a synergistic effect when using both methods simultaneously (47). 

**Mechanisms**

**Figure 1:**
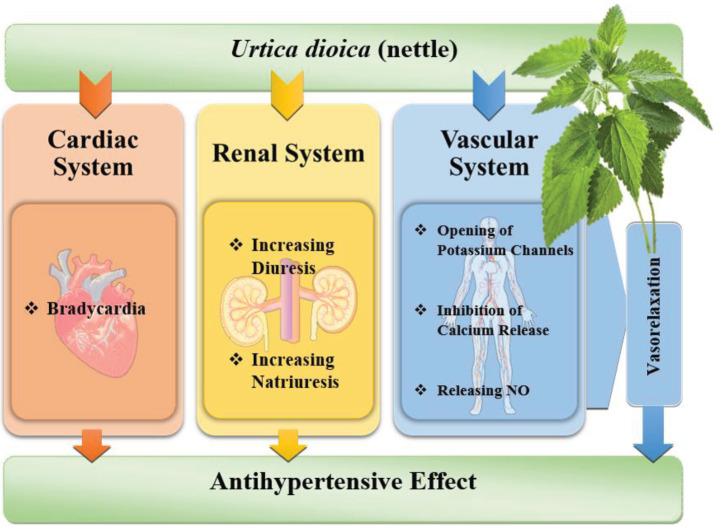
Different mechanisms of antihypertensive effects of nettle

The hypotensive effect of nettle is through several mechanisms suggested in different studies. Nettle could produce a vasorelaxant effect mediated by the release of endothelial NO. Elevated NO is associated with releasing cyclic guanosine monophosphate (cGMP) which leads to a hypotensive effect. Also, the opening of potassium channels and a negative inotropic action are considered to be helpful in this matter (48). The inhibitory effect on calcium ion moments through voltage-dependent channels is another mechanism causing the antihypertensive properties of nettle (42). The increase of diuresis and natriuresis are other reasons suggested for the lowering effect of nettle on blood pressure (38). Also, in an animal study, the dose-dependent bradycardia in an isolated heart was one of the mechanisms considered as the reason for the hypotensive activity of nettle (43). Different mechanisms that are important in the antihypertensive effects of nettle have been shown in Figure 1. Also the animal and clinical studies that show the antihypertensive effects of nettle have been summarized in Table 1. 

**Anti-hyperlipidemic activity**

Atherogenic dyslipidemia is a condition of three risk factors: Increased blood concentrations of LDL (small, dense low-density lipoprotein particles), decreased HDL (high-density lipoprotein particles), and increased TG (triglycerides) (49). A high level of serum LDL delivers cholesterol to the artery walls which leads to atherosclerosis, whereas HDL leads cholesterol from the tissues to the liver for catabolism (reverse cholesterol transport). Thus, HDL is known to have anti-atherogenic effects (50). Nowadays, herbal products are an important part of the treatment of dyslipidemia due to more popularity and safety and are easy to earn. Consumption of medicinal plants along with currently available chemical drugs could improve the management of hypertriglyceridemic patients (51). According to animal and human studies, there are several experiments in which by lowering levels of LDL and elevating levels of HDL, nettle is proven to be beneficial in the management of hyperlipidemia.

**Animal studies**

The influence of a herbal composition including nettle leaves (100 mg/kg) and burdock (*Arctium lappa*) roots extracts (25 mg/kg) on lipid metabolism has been investigated in 90 male diabetic rats in a 10-day trial. As a result hypertriglyceridemia and lipoperoxidation were effectively decreased (52). Nettle extract has caused hypocholesterolemic effects in male rats with high cholesterol diet in a 4-week period who received nettle extract (100 or 300 mg/kg) or lovastatin (10 mg/kg) as the control group (53). Daher *et al*. used the aqueous (150 mg/kg/day) and petroleum ether (20 mg/kg/day) extract of nettle for rats with a normal or high-fat diet in a 30-day trial. As expected, the levels of cholesterol, LDL, and LDL/HDL ratio were decreased which caused the blood lipid profile to improve (54).

In the model of diabetic rats induced by streptozotocin (STZ), the diet containing 15% (w/w) of a herbal mixture with 12 medicinal plants including nettle demonstrated beneficial effects on lipid profile (55). Mehran *et al*. suggest the helpful role of nettle in improving lipid metabolism by lowering serum cholesterol, LDL, LDL/HDL ratio, and TG. In his experiment, the effects of *U. dioica *and *Lamium album* extracts (100 mg/kg/day) on blood lipid profiles were compared in diabetic rats induced by STZ in a 28-day interval. A remarkable decrease in serum cholesterol level on the final day of treatment with both plant extracts was observed. Also, the serum LDL, LDL/HDL ratio, and TG level were lowered while the serum HDL level was elevated in rats treated with both plant extracts separately. Findings showed the decrease in serum TG in rats exposed to nettle extract was more significant than the other group, therefore *U. dioica *extract seems to be more effective in improving TG and fat metabolism than *L. album* extract (56). 

The effect of nettle extract on blood lipid profile has also been evaluated by Das *et al*. In this study, diabetic rats induced by STZ received an aqueous extract of nettle (1.25 g/kg). After 4 weeks, a significant reduction in the level of cholesterol was observed, and similar to earlier studies HDL level was found to be increased (57). In another study, diabetic rats induced by alloxan, received the aqueous extracts of *U. dioica *leaves, *Peganum harmala *seeds, and *Rhus coriaria* fruits either alone or in a triple mixture of 200 mg/kg/day. All the extracts caused a significant reduction in the level of LDL. However, the TG level was decreased only by treatment with nettle and the triplex mixture (58).

**Human studies**

A double-blind, randomized clinical trial with 50 diabetic women showed that 8-week treatment with a hydroalcoholic extract of nettle (5 ml TDS) could decrease the TG level and also increase HDL level in the intervention group compared to patients using the placebo. However, no significant differences in LDL level and plasma cholesterol were observed (59). In a similar single-blind randomized clinical trial, the hydroalcoholic extract of nettle (100 mg/kg/day) was consumed by diabetic patients for 8 weeks. The extract lowered levels of atherogenic indexes and TG and also elevated levels of HDL, at the end of the trial (46). In an 8-week, semi-experimental study, the effect of nettle supplementation and aerobic training on the lipid profile of diabetic patients has been measured. It was concluded that aerobic training along with the consumption of nettle powder is effective in increasing HDL and therefore controlling the lipid profile in type II diabetic patients (60). 

The effect of a herbal composition including nettle on lipid profiles of diabetic patients has been investigated in an unblinded, prospective interventional study. 119 patients received the composition (1 g TDS) in addition to their usual medications. After 12 weeks, their lipid condition showed decreased levels of mean total cholesterol and mean serum TG with good tolerability (61). It is indicated that nettle could also control obesity and its complications. Via a quasi-experimental study, the effect of 8 weeks of aerobic training and hydro-alcoholic extract of nettle (8 mg/day) has been investigated by measuring levels of inflammatory Apelin and high sensitivity C-reactive protein (hs-CRP) plasma in obese women. As a result, the levels of hs-CRP and Apelin were significantly decreased in the test group which points to the effective role of nettle extract along with aerobic exercise in controlling body fat percentage and obesity (62). Different animal and clinical studies that show the antihyperlipidemic effects of nettle have been summarized in Table 2.

**Mechanisms**

It is reported that nettle’s root extract could decrease 3-hydroxy-3-methyl-glutaryl-CoA (HMG-COA) reductase activity which causes a lowering effect on total cholesterol and plasma LDL levels in rats (63). Phenolic compounds and especially a group of flavonoids in nettle seem to have anti-oxidant activity (64). Therefore it has an important role in stabilizing lipid peroxidation (65). Quercetin is one of the flavonoid compounds in nettle. It is suggested that the consumption of quercetin could decrease total cholesterol and increase HDL cholesterol (66). Another mechanism considered is the effect of nettle on activating the peroxisome proliferator-activated receptor (PPAR). This causes a rise in the oxidation of fatty acids in the liver, which is associated with large reductions in serum lipids and adipose tissue mass (61).

**Anti-diabetic activity**

Diabetes mellitus is a common disorder worldwide. The latest calculations indicate that in 2017, 425 million people suffered from diabetes and this estimation is expected to rise to 629 million by 2045 (67). This epidemic disease has many complications including nephropathy, retinopathy, neuropathy, and diabetic foot. It could even lead to life-threatening conditions such as coronaropathy, stroke, and heart failure (68). Since having medicinal plants as remedies in patients with diabetes is common, the level of awareness in their usage among diabetic patients and their efficacy should be increased. In a survey among diabetic patients, it is demonstrated that 31% of interviewed subjects used herbal remedies daily (69). To prevent hyperglycemia, lifestyle and dietary modifications should be maintained in addition to single or combination drug therapies. However, there are still limitations in the use of pharmaceuticals to restore normal blood glucose homeostasis. Based on Mehri *et al*.’s review and Sarkhail, most of the studies are in favor of the benefits of  *U. dioica *in controlling hyperglycemia (70, 71). It is suggested that different compounds in nettle such as polyphenols, triterpenes, sterols, ﬂavonoids, and lectin could be responsible for its anti-diabetic features (72). 

***In vitro*** **studies**

It is stated that more than 400 plants across the world are beneficial in the treatment of diabetes, including nettle. According to research projects done in this matter, there are several mechanisms suggested on the anti-diabetic features of nettle (73). A study designed to determine the possible mechanisms of hypoglycemic effects of nettle has been conducted on RIN5F (model cell line with the ability to control the synthesis, storage, and secretion of insulin) rat pancreatic β cells and human muscle cells. The cultures were divided into two groups: One group had concentrations of 50, 100, and 200 μg/ml of alcoholic extract of nettle, and the other group had the same concentrations of extract plus insulin. After 60, 120, and 180 min, the mean glucose level was measured but it did not change significantly. It was observed that nettle extract couldn’t increase insulin sensitivity in muscle cells and/or increase insulin secretion from rat pancreatic β cells. Therefore, it is concluded that the hypoglycemic effects of nettle were not through the suggested mechanisms (74).

The major part of the human diet is carbohydrates. Pancreatic enzymes such as α-amylase help the chemical breakdown of digestible carbohydrates into monosaccharides in the small intestine. Eventually, carbohydrates are absorbed from the intestinal lumen into the blood circulation. The strong α-amylase inhibitory effect is suggested to be one of the therapeutic approaches of nettle’s antidiabetic effects (75). To confirm this matter, Rahimzadeh *et al*. incubated leaf aqueous extracts of *U. dioica *and *Juglans regia *with an enzyme-substrate solution and used acarbose as the standard inhibitor in measuring the enzyme activity. Nettle’s extract showed a 60% competitive inhibition by the concentration of 2 mg/ml (76). Another anti-hyperglycemic effect of nettle is its α-glucosidase inhibitory activity which was investigated in an *in vitro *study. Other plants such as *Taraxacum officinale*, *Viscum album*, and *Myrtus communis *were also examined and showed different potent α-glucosidase inhibitory activities (77). Not only *U. dioica *could potentiate insulin’s activity and enhance the utilization of glucose (78), but also one of its active fractions, separated by molecular sieve column chromatography, enhances glucose uptake by creating glucose permeable pores (79). Using molecular Docking with Molecular Operating Environment Software (MOE), it was shown that phenolic compounds of nettle could form more stable complexes with dipeptidyl peptidase 4 (DPP-4), α-amylase, and β-glucosidase (the main enzymes responsible for causing type 2 diabetes mellitus) than the original ligands. Therefore, it can be concluded that nettle’s phenolic compounds may be promising α-amylase and β-glucosidase inhibitors (80).

**Animal studies**

The anti-glycemic effect of nettle leaves extract was investigated in Wistar rats divided into two groups of STZ- induced diabetic rats and normal ones. They were divided into 4 groups which received a single dose of hydroalcoholic nettle extract either 500 mg/kg orally, 1000 mg/kg orally, 25 mg/kg intraperitoneally (IP), or 50 mg/kg (IP). As a result, diabetic rats showed a dose-dependent glucose-lowering effect with a stronger response in IP injected models. This effect was persistent for up to 48 hr. However, the normal rats did not show a significant hypoglycemic effect (81). 

In a 4-week investigation, the effect of nettle has been investigated on serum glucose and insulin resistance in model rats treated with fructose. The test group received 40-60 ml of nettle extract during the experiment. As a result, the subjects showed a significant decrease in serum glucose compared with the control group. Therefore it is concluded that nettle could decrease insulin resistance in rats treated with fructose (82). In a similar experiment by Sasan *et al*. after 8 weeks of treatment of subject rats with 21% fructose in water and 10% of aqueous extract of nettle for another 8 weeks, the blood and urine glucose was reduced in comparison with the high fructose-fed control group (83). It was shown that the aqueous extract of nettle (250 mg/kg) with the perfusion rate of 0.53 ml/min for 2 hr could decrease the intestinal glucose absorption in the jejunum of model rats. In presence of nettle, the glucose absorbed in 2 hr *in situ* on the jejunum segment was significantly less than that of the control group (84). The ethanolic extract of nettle evidently could influence the gene expression of glucose transporter 2 (GluT2) in the livers of diabetic mice induced by alloxan. In an 8-day experiment, the animals received nettle extract (150 mg/kg) intraperitoneally. The findings confirmed the effect of nettle extract on increasing GluT2 gene expression in the liver of diabetic mice which caused a hypoglycemic effect (85). 

Farzami *et al*. suggest that the hypoglycemic effect of nettle is due to its enhancement of insulin secretion by Langerhans islets. Furthermore, it could increase the insulin content of blood sera in normal and STZ-induced diabetic rats (86). In a 4-week experiment, diabetic rats induced by STZ, received nettle extract (12.5 ml/kg/day) via intragastric gavage. As a result, the serum insulin levels increased significantly and caused a reduction in blood glucose. Also, the islet volumes and β-cell numbers were significantly recovered, although the mean pancreatic β-cell volumes in the diabetic rats were not affected (87). A similar investigation was also performed to prove this matter via a randomized clinical trial on STZ-induced diabetic rats. In this 5-day investigation, the subjects received a hydroalcoholic extract of nettle (100 mg/kg/day) intraperitoneally. The results exhibited the protective activity of nettle extract on β-cells as well as its hypoglycemic effect in diabetic rats after 5 weeks (88, 89). In an 8-week study, STZ-induced diabetic rats were administered 15 mg/kg/day aqueous or ethanolic extract of nettle. At the end of the experiment, the diabetic rats in the control group showed injuries in pancreas tissue whereas the nettle-treated diabetic rats had slight rearrangements of islets (90). Combined herbal formulations containing nettle have also been effective for hyperglycemia treatment. In a 7-day study, alloxan-induced diabetic mice were treated with a single dose (20 mg/kg) of ethanolic herbal extract containing nettle via an esophageal tube. As predicted, decreased levels of blood glucose and fructosamine were observed (91). 

In an experiment on rabbits, test subjects were treated with a decoction of nettle followed by an injection of 50% dextrose solution to induce temporary hyperglycemia. The blood glucose was measured every 60 min for 5 hr. Unexpectedly, the result showed a slight increase in blood glucose in the test group (92). Similar to this result was the work of Swanston-Flat *et al*. who tried to evaluate the effects of different plants in glucose homeostasis in STZ-induced diabetic mice. Nettle was administered in the diet (6.25% by weight) for 28 days but at the end of the study, the diabetic condition was aggravated (93).

**Human studies**

Khajeh Mehrizi *et al*. have conducted a clinical trial with type 2 diabetic patients to evaluate the effect of nettle on glycemic control and insulin resistance in 8 weeks. The patients randomly consumed 100 mg/kg/day extract of nettle or placebo. At the baseline and the end of the trial, the levels of fasting blood glucose and insulin resistance indices including insulin concentration, insulin resistance, insulin sensitivity, and b-cell function, were measured in each candidate. As a result of this study, although significant effects on insulin resistance indices were demonstrated, the level of fasting blood glucose was not affected (94). With similar conditions as the former study, Tarighat Esfanjani *et al*. conducted an 8-week experiment in which hydroalcoholic extract of nettle (100 mg/kg/day) was consumed by type 2 diabetes patients. As a result, the level of fasting blood glucose was reduced significantly (95). Also, a quasi-experimental study was conducted with type 2 diabetic patients who had a decoction of nettle (10 g/day) for 8 weeks. At the end of the trial, blood glucose and body weight decreased significantly in the nettle-consumed group. The findings of this study conclude that consumption of nettle herbal tea could improve cardiovascular function and control blood glucose in diabetic patients (96). 

It is suggested that nettle leaves have anti-glycemic effects through their α-glucosidase inhibitory agents, PPAR-γ agonistic, and insulin secretory properties. Thus, in a randomized double-blind placebo-controlled clinical trial, the efficacy of taking nettle leaves’ extract in patients with type 2 diabetes mellitus needing insulin has been studied. In this study, one 500 mg capsule of nettle extract combined with oral anti-hyperglycemic drugs was taken every 8 hr for 3 months. As a result, the levels of fasting blood glucose, glycosylated hemoglobin (HbA1c), and 2 hr postprandial glucose (2hPPG) were significantly lowered compared with the controlled group (97). 

The effect of 8 weeks of aerobic training combined with supplementation of nettle (10 g/day) on glycemic parameters has been observed in a randomized clinical trial on diabetic men. The findings approved the glycemic control of nettle and a greater hypoglycemic effect in combination with nettle and aerobic training (98). In a similar study, diabetic females were studied and according to the findings, aerobic training and supplementation with nettle powder are effective in controlling blood glucose as complementarity methods (60). In several studies, the hypoglycemic effect of nettle in different herbal mixtures has been evaluated. One is the mixture containing *S. marianum *seeds, *U. dioica *leaves, and *Boswellia serrata *resin which was consumed by diabetic patients in a double-blind randomized placebo-controlled clinical trial for 90 days. The study confirmed the hypoglycemic effect of the herbal formulation) (99). As well as a former study, the glycemic control of another herbal mixture named “Glucolevel” was demonstrated in type 2 diabetic volunteers. This mixture contained a dry extract of leaves of *U. dioica*, *J. regia*, *O. europaea*, and *Atriplex halimus*. The patients received 3 Glucolevel tablets daily for 4 weeks and their glucose-homeostasis was regulated at the end of the trial) (100). 

Diabetic patients who receive nettle as an herbal supplement should be monitored regularly for their renal and hepatic function. Although there is no evidence proving the direct toxicity of nettle on kidneys and liver, high-profile studies should be planned for more information on toxic doses (71). Different animal and clinical studies that show the anti-diabetic effects of nettle have been summarized in Table 3. 

**Mechanisms**

According to the study by Mehi *et al*. the effects of nettle on reducing serum glucose could be categorized into two groups of pancreatic and extra-pancreatic. Impacts on β-cells and insulin release are included in pancreatic pathways while the actions affecting glucose homeostasis are in extra-pancreatic routes (70). Several mechanisms have been considered for the antidiabetic properties of nettle extract. One is via increasing insulin secretion by Langerhans islands and increasing the insulin content of blood sera (86). Nettle could also potentiate insulin activity and elevate the utilization of glucose (78). Furthermore, it enhances glucose uptake by creating glucose-permeable pores) (79). 

*In vitro* studies proposed the inhibitory effect of nettle on α-glucosidase as the reason for blood glucose-lowering activity (77), as well as its α-amylase inhibition activity (75). On top of that, in presence of nettle, the amount of intestinal glucose absorption in the jejunum is decreased (84). Moreover, nettle prevents islet atrophy, regenerates pancreatic β-cells, and restores plasma insulin levels which leads to hypoglycemia (87). Nettle extract could repair pancreatic tissues of diabetic rats by its effect on the size and number of the islets and histological parameters (90). 

**Figure 2:**
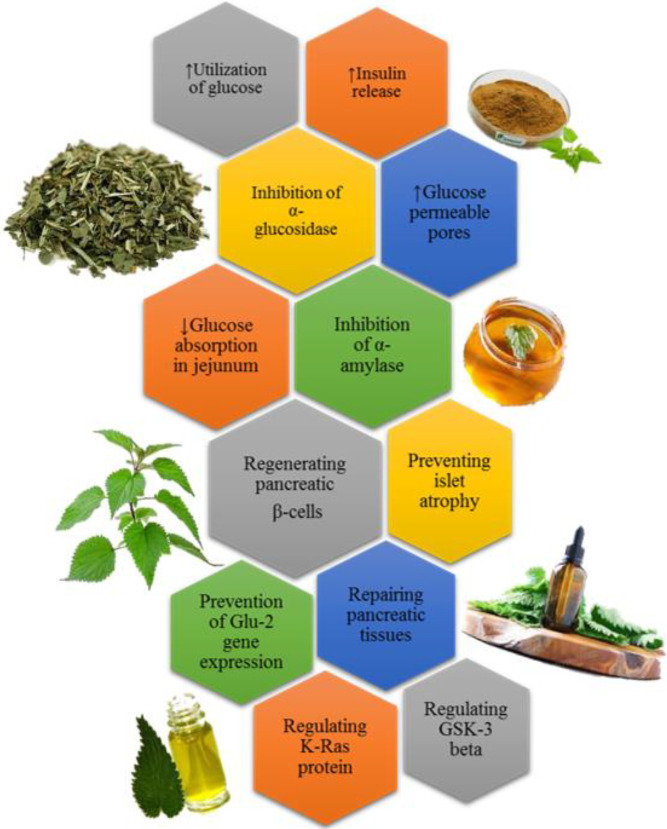
Different mechanisms of antihyperglycemic effects of nettle

Flavonoids present in nettle improve the blood glucose indexes via their anti-oxidant activity (101). Also, tannin and carotenoids, as constituents of nettle could improve blood glucose indexes (65). Through these anti-oxidant characteristics, nettle could rebuild β-cells in the pancreas (89). Evidence has also shown the preventive effect of nettle extract on Glut2 gene expression in the liver of diabetic mice which is another approach to its antidiabetic effect (85). Furthermore, hydroalcoholic extract of nettle could improve blood glucose level by regulating glycogen synthase kinase 3 beta (GSK-3 beta) and K-Ras protein (102). Different mechanisms that are important in the antihyperglycemic effects of nettle have been shown in Figure 2.

**Table 1.** The summary of animal and clinical studies regarding anti-hypertension effects of nettle and related mechanisms

***Type of Study***	***Type of animal***	***Dosage of Nettle Extract***	***Duration of Study***	***Induction of hypertension***	***Main Outcomes***	***Mechanisms***	***Ref.***
***Animal***	rat	4 mg/kg/h or 24 mg/kg/h	1.25 hr	0.9% NaCl	↓arterial blood pressure	↑diuresis and natriuresis	(38)
	rat	200 mg/kg/day	4 weeks		↓cardiac index ↓systolic and diastolic blood pressure		(37)
	rat	1, 3, 10, 30 and 50 mg/kg	6 weeks	high-salt (8 % NaCl) diet	↓mean arterial pressure	calcium channel blocking effects, nitric oxide (NO)-mediated vasorelaxation	(42)
	rat	1 and 2 g/l	30 min		↓blood pressure	bradycardia	(43)
***Human***	___	300 mL/day	16 weeks	___	↓blood pressure		(103)
	___	100 mg/kg/day	8 weeks	___	↓systolic blood pressure		(46)

**Table 2:** The summary of animal and clinical studies regarding anti-hyperlipidemic effects of nettle and related mechanisms

***Type of Study***	***Type of animal***	***Dosage of Nettle Extract***	***Duration of Study***	***Induction of hyperlipidemia***	***Main Outcomes***	***Mechanisms***	***Ref.***
***Animal***	rat	100 mg/kg	10 days	Diet with increased fat content (30%)	**↓**Hypertriglyceridemia, **↓**lipoperoxidation		(52)
	rat	100 or 300 mg/kg	4 weeks	10 ml/kg/day of a cocktail (1 L peanut oil, 100 g cholesterol, 30 g propylthiouracil,100 g cholic acid)	hypocholesterolemic effects		(53)
	rat	150 mg/kg/day	30 days	diet enriched with coconut oil as 5% w/w	**↓**cholesterol, LDL, and LDL/HDL ratio **↓**blood lipid profile		(54)
	rat	15% (w/w)	4 weeks		**↓**lipid profile		(55)
	rat	1.25 g/kg	4 weeks		**↓**cholesterol ↑HDL		(57)
	rat	triple mixture of 200 mg/kg/day	4 weeks		**↓**LDL, TG		(58)
	rat	100 mg/kg/day	28 days		**↓**serum cholesterol, LDL, LDL/HDL ratio, and TG ↑HDL	improving lipid metabolism	(56)
***Human***	___	5 ml TDS	8 weeks	___	**↓**TG ↑HDL		(59)
	___	100 mg/kg/day	8 weeks	___	**↓**atherogenic indexes and TG ↑HDL		(46)
	___		8 weeks	___	↑HDL		(60)
	___	1g TDS	12 weeks	___	**↓**mean total cholesterol and mean serum TG	oxidation of fatty acids via activating PPAR	(61)
	___	8 mg/day	8 weeks	___	**↓**body fat percentage	**↓**hs-CRP and Apelin	(62)

PPAR: peroxisome proliferator-activated receptor

**Table 3.** The summary of animal and clinical studies regarding anti-diabetic effects of nettle and related mechanisms

***Type of Study***	***Type of animal***	***Dosage of Nettle Extract***	***Duration of Study***	***Induction of Diabetes***	***Main Outcomes***	***Mechanisms***	***Ref.***
***Animal***	rats	40-60 ml/day	4 weeks	Fructose (66%)	**↓**serum glucose	**↓**insulin resistance	(82)
	rats	50, 100 or 200 mg/kg/day	2 weeks	Fructose (10%)	**↓**serum glucose	↓FIRI	(104)
	rats	10%	8 weeks	Fructose (21%)	↓serum and urine glucose		(83)
	rats	250 mg/kg	2 hr	Alloxan (120 mg/kg/day)	**↓**serum glucose	↓intestinal glucose absorption	(84)
	mice	150 mg/kg	8 days	Alloxan (200 mg/kg/day)	↓serum glucose	prevented GluT2 gene expression	(85)
	mice	20 mg/kg	1 week	Alloxan (65 mg/kg)	↓serum glucose, ↓fructosamine		(91)
	rabbits	4 ml/kg	5 hr	Dextrose (50%, 4 ml/kg)	↑serum glucose		(92)
	rats		1 week	STZ (40 mg/kg)	↓serum glucose	↑insulin secretion	(86)
	rats	12.5 ml/kg/day	4 weeks	STZ (50 mg/kg)	↓serum glucose recovered islet volumes and β-cell numbers	↑insulin secretion, preventing islet atrophy and/or regenerating pancreatic β-cells	(87)
	rats	100 mg/kg/day	5 weeks	STZ (80 mg/kg)	↓serum glucose	protective effect on β-cells	(88, 89)
	rats	15 mg/kg/day	8 weeks	STZ (50 mg/kg)	slight to moderate rearrangement of pancreatic islets	protective effect on pancreatic islets	(90)
	rats	1.25 g/kg	4 weeks	STZ (90 mg/kg)	↓fasting serum glucose		(57)
	rats	100 mg/kg/day	5 days	STZ (80 mg/kg)	↓serum glucose	proliferation of β-cells	(34)
	rats	100 mg/kg/day	4 weeks	STZ (60 mg/kg)	↓serum glucose		(56)
	rats	0.40-0.60 ml/day	4 weeks	STZ (65 mg/kg)	↓fasting serum glucose		(33)
	mice	6.25%	4 weeks	STZ (200 mg/kg)	↑serum glucose		(93)
	rats	625 mg/kg, 1.25 g/kg	4 weeks	STZ (50 mg/kg)	↓serum glucose ↑insulin resistance ↑insulin sensitivity	↑insulin secretion, ↑glucose uptake, regeneration of β-cells, ↓β-cell damage	(105)
	rats	100 mg/kg	4 weeks	STZ (60 mg/kg)	↓serum glucose	↓GSK-3 beta level ↑K-Ras	(102)
	rats	500, 1000 mg/kg orally, 25, 50 mg/kg IP	single dose	STZ	hypoglycemic effect with a stronger response in IP models		(81)
***Human***	___	100 mg/kg/day	8 weeks	___	no significant effect on fasting blood glucose	↓insulin resistance	(94)
	___	100 mg/kg/day	8 weeks	___	↓fasting blood glucose		(95)
	___	10 g/day	8 weeks	___	↓blood glucose, ↓body weight		(96)
	___	500 mg TDS	3 months	___	↓fasting blood glucose, ↓2hPPG, ↓HbA1c	inhibition of α-glucosidase, PPAR-γ agonistic properties, ↑insulin secretion	(97)
	___	10 gr/day	8 weeks	___	↓blood glucose		(98)
	___		8 weeks	___	↓mean serum fasting blood glucose, ↓glycosylated hemoglobin		(60)
	___	1 capsule (200mg/600mg) TDS	90 days	___	↓blood glucose		(99)
	___	3 Glucolevel tablets/day	4 weeks	___	regulated glucose-homeostasis		(100)

FIRI: Fasting insulin resistance index, GluT2: Glucose transporter 2, STZ: Streptozotocin, IP: Intraperitoneally, 2hPPG: 2 hr postprandial glucose, HbA1c: Hemoglobin A1C, PPAR: Peroxisome proliferator-activated receptor, GSK-3 beta: Glycogen synthase kinase 3 beta, TDS: 3 times a day

## Conclusion

The present review has evaluated a variety of *in vitro*, *in vivo*, and clinical studies regarding the beneficial effects of nettle in treating different symptoms of metabolic syndrome. According to this evaluation, most of the studies were related to antidiabetic aspects of nettle. This valuable plant has exhibited various positive effects on hypertension, hyperlipidemia, and diabetes. The hypotensive features of nettle are through various pathways such as vasorelaxation. Nettle is also a valuable plant in treating hyperlipidemia as it stabilizes lipid peroxidation and increases the oxidation of fatty acids in the liver. The effects of nettle on lowering blood glucose could be the result of pancreatic or non-pancreatic pathways. Several mechanisms for the potential effects of nettle have been summarized in this review, however, further detailed studies are required to underlie the molecular mechanisms. Taken together, nettle can be a useful herbal remedy in different components of metabolic syndrome.

## Conflicts of Interest

The authors have no conflicts of interest to declare.
